# Leptomeningeal Metastases from Solid Tumors: Recent Advances in Diagnosis and Molecular Approaches

**DOI:** 10.3390/cancers13122888

**Published:** 2021-06-09

**Authors:** Alessia Pellerino, Priscilla K. Brastianos, Roberta Rudà, Riccardo Soffietti

**Affiliations:** 1 Department of Neuro-Oncology, University and City of Health and Science Hospital, 10126 Turin, Italy; rudarob@hotmail.com (R.R.); riccardo.soffietti@unito.it (R.S.); 2 Massachusetts General Hospital Cancer Center, Harvard Medical School, Boston, MA 02115, USA; pbrastianos@mgh.harvard.edu; 3 Department of Neurology, Castelfranco Veneto and Brain Tumor Board Treviso Hospital, 31100 Treviso, Italy

**Keywords:** ALK-rearranged NSCLC, blood–brain barrier, BRAF-mutated melanoma, EGFR-mutated NSCLC, HER2-enriched breast cancer, immunotherapy, leptomeningeal metastases, liquid biopsy, triple-negative breast cancer

## Abstract

**Simple Summary:**

Leptomeningeal metastases are a devastating complication of solid tumors with poor survival, regardless of the type of treatments. The limited efficacy of targeted agents is due to the molecular divergence between leptomeningeal recurrences and primary site, as well as the presence of a heterogeneous blood-brain barrier and blood-tumor barrier that interfere with the penetration of drugs into the brain. The diagnosis of leptomeningeal metastases is achieved by neurological examination, and/or brain and spinal magnetic resonance, and/or a positive cerebrospinal fluid cytology. The presence of neoplastic cells in the cerebrospinal fluid examination is the gold-standard for the diagnosis of leptomeningeal metastases; however, novel techniques known as “liquid biopsy” aim to improve the sensitivity and specificity in detecting circulating neoplastic cells or DNA in the cerebrospinal fluid. Targeted therapies and immunotherapies have changed the natural history of metastatic solid tumors, including lung, breast cancer, and melanoma. Targeting actionable mutations, such as epidermal growth factor receptor-mutated and anaplastic lymphoma kinase-gene rearranged in lung cancer, human epidermal growth factor receptor 2-positive breast cancer, and BRAF-mutated melanoma, have led to encouraging results also in leptomeningeal metastases. On the other hand, immunotherapy or modified traditional chemotherapy are under investigation in LM from non-druggable tumors.

**Abstract:**

Leptomeningeal metastases (LM) from solid tumors represent an unmet need of increasing importance due to an early use of MRI for diagnosis and improvement of outcome of some molecular subgroups following targeted agents and immunotherapy. In this review, we first discussed factors limiting the efficacy of targeted agents in LM, such as the molecular divergence between primary tumors and CNS lesions and CNS barriers at the level of the normal brain, brain tumors and CSF. Further, we reviewed pathogenesis and experimental models and modalities, such as MRI (with RANO and ESO/ESMO criteria), CSF cytology and liquid biopsy, to improve diagnosis and monitoring following therapy. Efficacy and limitations of targeted therapies for LM from EGFR-mutant and ALK-rearranged NSCLC, HER2-positive breast cancer and BRAF-mutated melanomas are reported, including the use of intrathecal administration or modification of traditional cytotoxic compounds. The efficacy of checkpoint inhibitors in LM from non-druggable tumors, in particular triple-negative breast cancer, is discussed. Last, we focused on some recent techniques to improve drug delivery.

## 1. Introduction

Leptomeningeal metastases (LM) are defined as the infiltration of leptomeninges, including the pia mater, arachnoid and subarachnoid space, from a primary solid tumor. LM represent the third most frequent metastatic complication of the central nervous system (CNS) after brain metastases (BM) and epidural metastases [1], with an overall survival (OS) ranging from weeks to months, regardless of the type of treatment. In this regard, autopsy series have shown the presence of undiagnosed or asymptomatic LM in 19% of patients with solid tumors, of which 5% are an end-stage complication following systemic treatments [2]. Solid tumors with a significant risk of leptomeningeal recurrence are melanoma in 5–7% of patients [3], non-small-cell lung cancer (NSCLC) and breast cancer in 3–5%, respectively [4,5]. The incidence of LM is increasing due to the improvement of tools for diagnosis and monitoring, as well as the availability of more active targeted therapy to control systemic disease, while being less effective in CNS due to the presence of the blood–brain barrier (BBB). Here, we review the role of the BBB in regulating the penetration into the CNS of targeted therapy and immunotherapy and the diagnostic challenges in LM, including the role of magnetic resonance imaging (MRI), cerebrospinal fluid (CSF) cytology and liquid biopsy. Furthermore, we discuss the impact of targeted therapies in LM from solid tumors with actionable mutations, such as epidermal growth factor receptor (EGFR)-mutated and anaplastic lymphoma kinase (ALK)-gene rearranged NSCLC, human epidermal growth factor receptor 2 (HER2)-positive breast cancer (BC) and BRAF-mutated melanoma. Last, some evidence is provided on the impact of immunotherapy in LM from solid tumors without druggable mutations.

## 2. The Role of the Blood–Brain Barrier, Blood–Tumor Barrier and Blood–CSF Barrier in Drug Delivery

The BBB consists of endothelial cells (ECs) linked by tight junctions (TJs), which are surrounded by pericytes and astrocytic endfeet, that contribute to the integrity of the basal lamina. The surface of the basal lamina provides molecules that may activate multiple signaling pathways to maintain the CNS homeostasis and regulate the passage of molecules through the BBB [6]. ECs create a continuous, non-fenestrated barrier with a reduced number of pores that limit the vesicular trafficking and pinocytosis [7]. In general, molecules may cross the BBB by means of several mechanisms: (1) paracellular transport, which depends on physicochemical properties of molecules, such as molecular weight, lipophilicity and electrical charge, and remains limited to small lipophilic molecules (e.g., oxygen, caffeine) [8]; (2) transcellular transport characterized by a flow from the luminal side of the ECs to the abluminal side into the brain interstitium, using either vesicle-mediated transcytosis (receptor-mediated or adsorptive routes) or carrier-mediated transcytosis that are mainly used by small hydrophilic molecules (glucose, insulin, amino acids, albumin, infectious agents and neurotoxins) [9]. The main role of the BBB is to interfere with the penetration of exogen agents and toxins into the CNS. As for antineoplastic drugs, most small molecules and nearly 100% of large compounds have poor penetration through the BBB [10], resulting in a significant control of systemic disease, while CNS remains a frequent site of relapse [11,12]. The ATP-binding cassette transporters (ABC transporters) mediate the efflux toward the luminal space with the aim to clear brain parenchyma and CSF space from most antineoplastic compounds [13]. As ABC transporters are expressed either on ECs or astrocytes, microglia and neurons, the penetration through the BBB is not the only factor that impacts an adequate concentration of drugs into the brain parenchyma or CSF [14]. For instance, the multidrug resistant (MDR) ABC transporters, P-glycoprotein (P-gp or ABCB1), breast cancer resistance protein (BCRP or ABCG2) and multidrug resistance proteins (MRPs) affect the balance between influx and efflux and the therapeutic delivery of chemotherapy and targeted therapy [15], representing a barrier to overcome in order to improve drug concentrations in CNS.

During tumor progression in the CNS, BBB is disrupted and replaced with a dysfunctional interface represented by the blood–tumor barrier (BTB), which consists of tortuous vessels, an abnormal pericyte distribution and loss of astrocytic endfeet, leading to heterogeneous permeability to drugs, as well as a heterogeneous perfusion that contributes to an inadequate drug accumulation in tumor cells [16]. In this regard, pericytes present a different composition of desmin-positive subpopulations in BM/LM when compared with normal vessels of brain tissue [17]. Similarly, reactive astrocytes reduce the expression of the omega-3 fatty acid transporter for the docosahexaenoic acid (DHA) on ECs, which is necessary for neuronal function and neuroprotection, leading to the loss of the endfeet connection with the ECs [18]. Moreover, the increased expression of the sphingosine 1-phosphate receptor 3 (S1PR3) on reactive astrocytes determines a loss of the interaction with BTB via interleukin-6 (IL6) and CC chemokine ligand 2 (CCL2) secretion, resulting in a leakier and heterogenous permeability to drugs [19]. Importantly, the BBB/BTB interface may differ among BM/LM from different subtypes of solid tumors. For instance, the BTB of CNS recurrences from HER2-positive BC retains a higher expression of glucose transporter 1 (GLUT1) and BCRP compared with other molecular subtypes [20]. Preclinical models have shown that the small molecule lapatinib has a different distribution in BM from HER2-enriched BC and healthy brain tissue [21]. Table 1 displays some examples of BBB/BTB heterogeneity in preclinical and clinical studies in CNS metastases from solid tumors.

A further barrier that limits the penetration of compounds is represented by the blood–CSF barrier, which comprises the TJs between choroid plexus epithelial cells. Since the BBB and blood CSF barrier use different active transport mechanisms to regulate the passage of molecules, and CSF drug concentrations depend on the permeability of the blood–CSF barrier, drug delivery into CNS cannot be considered as a surrogate of drug concentrations in LM [9]. The absence of a reliable tool to determine whether drugs adequately cross the BBB is the main goal of future phase 0 trials with the aim to investigate the drug target effects, as well as the pharmacokinetic–pharmacodynamic features in an early clinical setting [33].

## 3. Pathogenesis of Leptomeningeal Metastases

Four main routes favor the leptomeningeal dissemination from solid tumors: (i) hematogenous spread through arterial vessels; (ii) venous circulation through bridging venous or the Batson’s plexus, which is a network of veins that connect the deep pelvic and thoracic veins and drain blood from the urinary bladder, breast and prostate to the internal vertebral venous plexuses; (iii) the neural route through cranial nerves or spinal roots; and (iv) from the brain parenchyma by contiguity. The arterial and venous routes are considered the major routes used by NSCLC and BC to spread, while the perineural route has been associated with melanoma [34,35]. Some iatrogenic dissemination to leptomeninges may occur after surgery of BM, especially in the posterior fossa, using a piecemeal compared with en bloc tumor resection or when access to the ventricular system is required [36]. Moreover, stereotactic radiosurgery (SRS), especially when treating a resection cavity, has been suggested to increase the risk of local LM up to 31%, although the data in this area are still limited and need to be explored [37,38].

New insights in the molecular mechanisms underlying LM development are emerging. Boire et al. reported that complement component 3 (C3), which is produced by tumor cells in the CSF, is overexpressed in LM models of NSCLC and BC. C3 interacts with the C3a receptor (C3aR) on the epithelial cells of the choroid plexus, perturbs the barrier function and allows the passage of mitogen factors, such as amphiregulin, that drive tumor growth in leptomeninges. When C3aR signaling is blocked using a specific antagonist, LM development is suppressed [39]. Similarly, Conrad et al. reported that matrix metalloproteinases (MMPs) type 9 and a disintegrin and metalloproteases (ADAMs) type 8–17 are markers of extracellular matrix degradation in CSF following leptomeningeal dissemination and blood–CSF barrier disruption, promoting the entry of tumor cells in subarachnoid space [40]. Furthermore, tumor cells gain some mechanisms for surviving in a CSF microenvironment with poor micronutrients. CSF samples from five patients with LM were analyzed using single-cell RNA sequencing, showing that tumor cells, but not macrophages, within the CSF express the iron-binding protein lipocalin-2 (LCN2) and its receptor SCL22A17. These macrophages produce inflammatory cytokines that stimulate LCN2 expression on tumor cells but do not generate LCN2 themselves. In mouse models of LM, when iron levels are reduced by chelation therapy, tumor cell growth is inhibited, suggesting that cancer cells survive in the CSF by outcompeting macrophages for iron [41]. Notably, Remsik et al. [42] used leptomeningeal derivatives of human breast and lung cancer to show that tumor cells in CSF may have a floating or adherent phenotype: the floating phenotype corresponds to disease in the CSF, while the adherent phenotype is enhancing on MRI. Tumor cells of the floating phenotype have a decreased proliferation rate, lower ATP content and are enriched of peculiar metabolic signatures, such as tricarboxylic acid cycle and electron transport chain signatures, resulting in a metabolic flexibility of LM cells in adapting to the limited glucose levels in the CSF. Furthermore, the floating cells disseminate into mouse leptomeninges earlier and are associated with a shorter survival in comparison with the adherent phenotype. Some studies have shown the development of LM from BC and NSCLC close to BM using murine models. Palmieri and Allen reported the ability of brain-metastatic MDA-MB-231 cells to generate both HER2-positive parenchymal and leptomeningeal disease from BC after intracardiac injection [43,44]. Recently, Dankner et al. [45] reported a different propensity to LM based on the pattern of invasion in BM (minimally invasive versus highly invasive), which is driven by the expression of the cold-inducible RNA-binding protein (CIRBP). These data suggest that specific molecular pathways are present in a subset of BM cells only and are involved in leading the invasion of leptomeninges. Another molecular mechanism associated with leptomeningeal spreading is the acquired resistance to first-generation targeted therapy. In this regard, Nanjo et al. displayed that the acquired resistance to gefitinib in LM from NSCLC is associated with an overexpression of MET proto-oncogene and a lack of T790 mutation [46]. In fact, T790 mutation has not been detected in LM or the CSF of patients pretreated with EGFR tyrosine kinase inhibitors (TKIs) [47,48], and Jiang et al. reported a low frequency (21%) of T790 mutation and a high prevalence (39%) of MET amplification in the CSF [49], arguing that MET amplification and absence of T790 mutation may be hallmarks of leptomeningeal invasion [50].

A further factor limiting the efficacy of targeted agents in LM may be the potential molecular divergence between primary tumors and CNS lesions. Brastianos et al. [51] demonstrated that specific genetic alterations were not found in the matched-primary tumor sample in 53% of BM from BC, NSCLC and renal cancer. However, spatially and temporally separated BM were more genomically homogeneous. BM shared PI3K/AKT/mTOR, CDK and HER2/EGFR mutations. Further investigations displayed that the amplification of MYC, YAP1 and MMP13 and the deletion of CDKN2A/B are frequent genetic aberrations in BM from NSCLC; however, it is unknown whether they play a key role in leptomeningeal dissemination [52]. Molecular divergence has also been reported in BM from BC. Approximately 16–22% of BM from HER2-negative BC have been reported to gain HER2 amplifications and EGFR overexpression [43,53], as well as PTEN loss [54], compared with the primary site. Overall, the molecular profiling of CNS recurrence and the primary tumor should be necessary to choose the most adequate treatment. However, surgery is not always feasible, especially in LM; thus, alternative techniques to predict molecular subtypes, such as liquid biopsy, need to be developed.

## 4. Diagnosis of Leptomeningeal Metastases

The diagnosis of LM is achieved by combining neurological evaluation, an MRI of the brain and spinal axis and/or the identification of tumor cells in the CSF, which is the gold standard for diagnosis. The European Association of Neuro-Oncology-European Society of Medical Oncology (EANO-ESMO) group has proposed a diagnostic flowchart that includes neurological symptoms, imaging and CSF cytology for diagnosis, treatment and follow-up of patients with LM from solid tumors [55]. The combination of these three items allows one to define the diagnosis of LM as type I with positive CSF cytology or type II (probable/possible) with typical MRI characteristics and neurological signs. Based on the MRI pattern, LM may be defined as linear (subtype A), nodular (subtype B), both (subtype C) or hydrocephalus (subtype D). Recently, the EANO-ESMO group has retrospectively reviewed 254 LM from solid tumors using the aforementioned guidelines, reporting a remarkable prognostic value in predicting OS. In particular, patients with type I have a shorter OS than type II LM. Concerning MRI findings, nodular disease negatively impacts survival in type II but not in type I LM. Lastly, the administration of either systemic or intrathecal therapy is associated with improved OS in type I, but not in type II LM, although this needs to be explored in bigger datasets and prospective trials. Overall, the EANO-ESMO LM classification is highly prognostic and has been recommended for stratification and design of clinical trials [56].

### 4.1. Neurological Symptoms

Symptoms of LM are typically multifocal reflecting the involvement of spinal cord and nerve roots in 60% of patients, cranial nerves in 35% and the cerebrum in 15%. The differential diagnosis includes symptoms associated with BM or other conditions, such as treatment-related toxicities or neurological paraneoplastic syndromes. Headache and nausea (66%), spinal and/or radicular symptoms (46%), diplopia, visual impairment and hearing loss (36%) are the most frequent symptoms of LM, while dysphagia, mental changes and seizures are late signs of encephalopathy, which correlate with poor outcome [34]. The Leptomeningeal Assessment in Neuro-Oncology (LANO) group has proposed a standardized assessment for the neurological examination with multiple domains, including gait, strength, sensation, vision, eye movement, facial strength, hearing, swallowing, level of consciousness and behavior, with the aim to be utilized by neurologists, neuro-oncologists, medical oncologists, nurses and physician assistants, but it needs to be prospectively validated [57].

### 4.2. Neuroimaging Assessment

**Figure 1:**
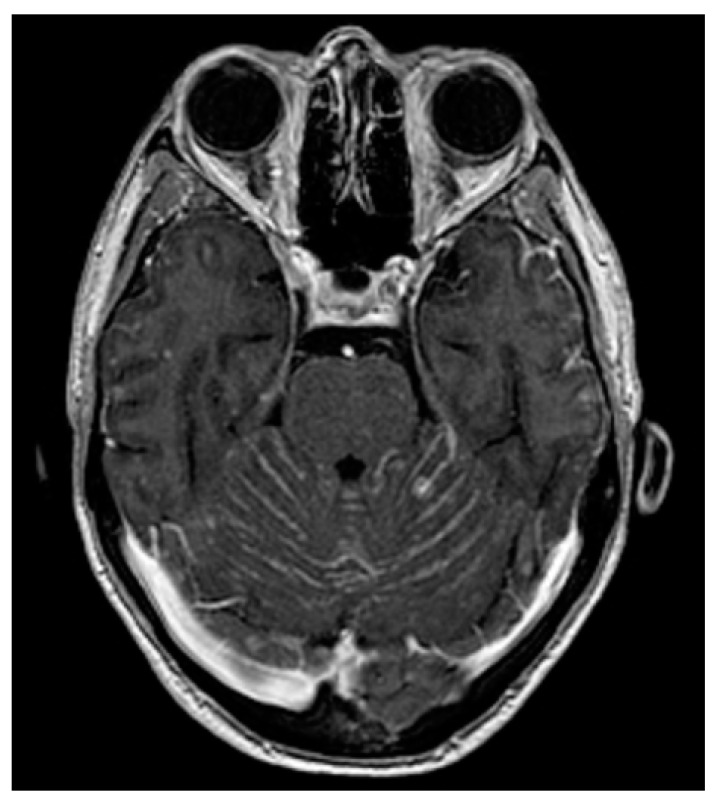
Cerebellar localizations of a HR-positive/HER2-negative breast cancer following CDK4/6 inhibitor palbociclib.

**Figure 2:**
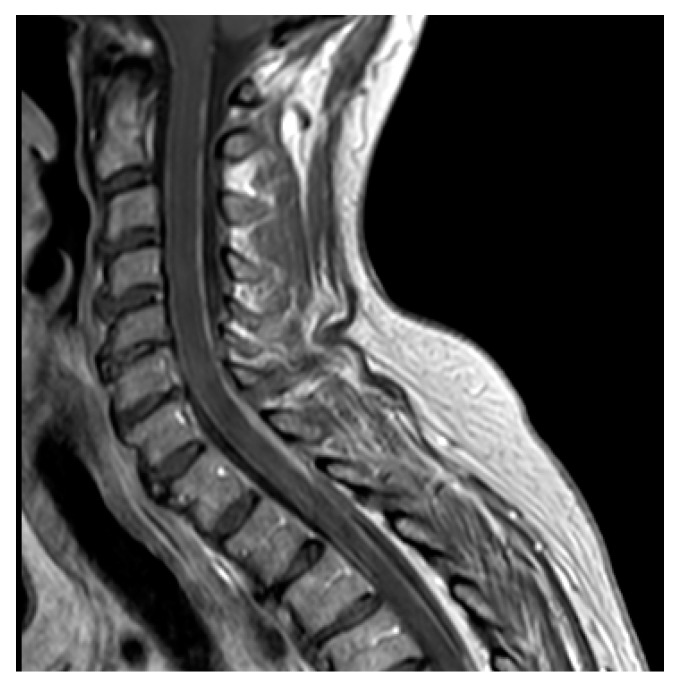
Cervical leptomeningeal carcinomatosis of an ALK-rearranged NSCLC after failure of the first- and second-generation ALK inhibitors (crizotinib and alectinib, respectively).

**Figure 3:**
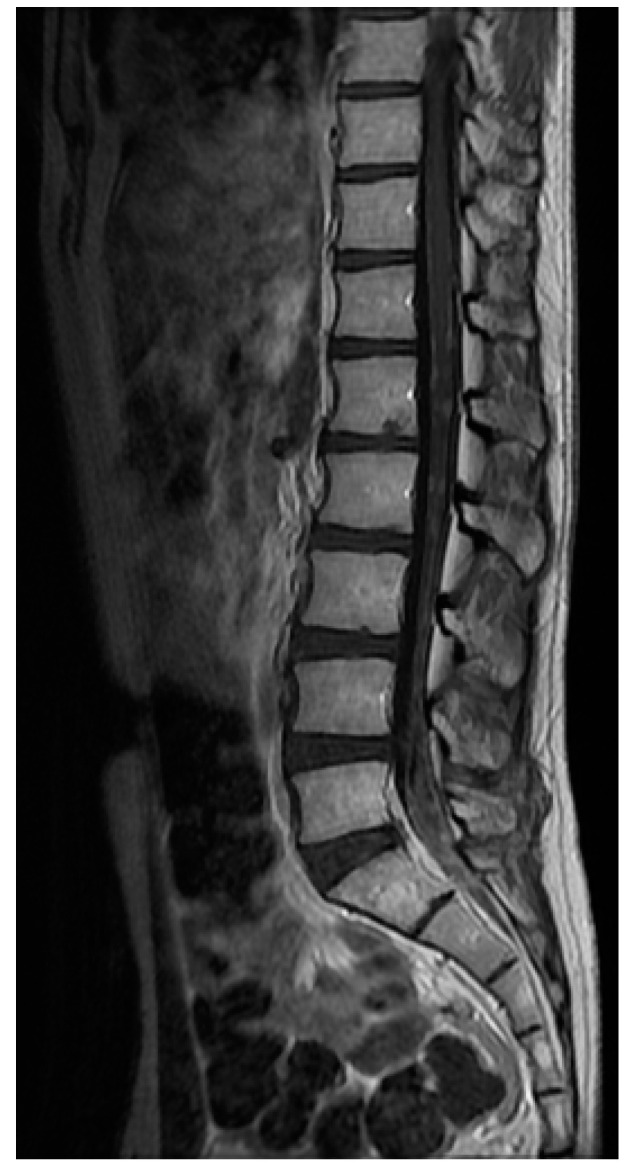
Leptomeningeal metastases of the cauda equina from a BRAFv600-mutated melanoma.

Brain and spinal MRIs are the current methods for the diagnosis of LM [55], which may present different patterns of enhancement, such as nodular, linear or curvilinear, as well as focal or diffuse features, with a significant inter-observer variability to classify the lesions [58] (Figure 1, Figure 2 and Figure 3). The integration of MRI findings and CSF cytology helps to better stratify patients: patients with type 2A or 2C nodular LM lesions have a worse OS compared with those with non-nodular disease [56]. Conversely, the presence of nodular LM is associated with an improved OS in patients with BM treated with surgery followed by adjuvant SRS when compared with diffuse linear LM [59,60,61]. Furthermore, the pattern of enhancement is not the unique radiological factor that impacts the outcome, but also the location of LM has been suggested to influence the prognosis. In fact, patients with only cranial involvement display a better outcome than those with both cranial and spinal LM [62].

### 4.3. CSF Cytology and Liquid Biopsy

The identification of neoplastic cells in CSF is the gold standard for the diagnosis of LM, and the presence of a positive CSF cytology is correlated with a worse OS [56]. However, the sensitivity of CSF cytology is limited to 44–67% at the first lumbar puncture and increases to 84–91% after repeated sampling [63,64,65,66,67,68]. Moreover, the presence of “suspicious” or “atypical” cells may impact the sensitivity and specificity of conventional CSF cytology [69]. Hence, some novel techniques have been developed to improve the detection of circulating tumor cells (CTCs) in the CSF using immunoflow cytometry with fluorescently labeled antibodies against membrane-bound tumor cell proteins, such as the epithelial cell adhesion molecule (EpCAM) for epithelial tumor cells [70], and human high molecular weight-melanoma-associated antigen (HMW-MAA/MCSP) or melanoma chondroitin sulfate proteoglycan (MCSP) and CD146 for melanoma [71,72]. The CellSearch platform is a method of EpCAM-based rare cell capture technology (RCCT), which uses an immunomagnetic CTC selection using EpCAM antibody conjugated ferroparticles, able to provide a quantitative assessment of cancer cells in a limited amount of CSF (3 cc). Some studies using immunoflow cytometry or an adapted CellSearch technique for searching for CTCs in the CSF report a sensitivity ranging from 75 to 100% and a specificity of 84–100% (Table 2). However, these studies have major limitations, including a small sample size, and mainly focused on NSCLC and BC. Data on sensitivity and specificity of the CellSearch platform based on immunofluorescence detection of HMW-MAA, CD45 and CD34 for patients with melanoma were reported in two patients only [72] due to the decision of the company to not pursue further development of the technique. Moreover, it is unknown whether immunoflow cytometry and CellSearch technology are comparable in terms of detecting CTCs. As epithelial tumor cells can lose EpCAM expression due to the transition to mesenchymal subtype [73] and HMW-MAA/MCSP expression on melanoma cells is only found in 85%, both EpCAM and HMW-MAA/MCSP assays could fail in detecting CTCs. In light of that, the CSF cytology may help to increase the specificity when the CTC assay is negative. An advantage of detecting CTCs is to provide data on tumor burden, while CSF cytology cannot provide quantitative information. Some small studies have demonstrated a correlation of quantification of CTCs in CSF and the prediction of survival in LM [72,74,75], but the cutoff value needs to be validated in larger and prospective cohorts. Another advantage of CTCs is to isolate single CTCs for searching genetic aberrations that are shared by primary solid tumors. In this regard, CTCs from the CSF of patients with LM from EGFR-mutated or ALK-rearranged NSCLC have a highly concordant molecular profile (89.5%) with a paired primary tumor [76]. Conversely, Magbanua and Li performed a genomic sequencing on isolated BC cells in the CSF of patients and reported some shared mutations with the primary BC, as well as new mutations, suggesting a molecular divergence in LM [77,78]. Importantly, some of these distinct mutations, such as syndecan-1 and MUC-1 overexpression, have been correlated to leptomeningeal invasion [79].

Typically, CSF is enriched with cell-free tumor DNA (ctDNA), which can be extracted and analyzed using digital PCR for a limited number of genes or undergo whole-exome sequencing based on the clinical question. Of note, the use of ctDNA from CSF has been reported to be more sensitive compared with the blood for the detection of druggable mutations in BM from solid tumors. In this regard, EGFR, PTEN, ESR1, FGFR2 and ERBB2 were more frequently detected in CSF ctDNA than in blood in a cohort of NSCLC and BC [86] as well as in patients with melanoma and negative CSF cytology [87]. Pentsova et al. found targetable mutations in the CSF of 20/32 patients (63%) with BM, while no mutations were detected in patients without CNS involvement [88]. Because cellular material in the CSF contains both normal and cancer cell DNA, extracting from the acellular material gives a significant amount of DNA from tumor cells [88]; thus, the detection of ctDNA in LM holds promise (Table 3). Momtaz et al. extracted and sequenced ctDNA from three out of three patients with confirmed radiological LM from BRAF-mutated disease, including melanoma [87]. Marchiò et al. isolated ctDNA with KRAS mutation from the CSF of two patients with LM from NSCLC, but not in blood [89]. In addition, Swinkles et al. found a mutation of KRAS using PCR sequencing in LM of patients with a negative CSF cytology, suggesting that the early detection of ctDNA may influence the prognosis [90]. CtDNA was also successfully detected in 11 (100%) [91] and 28 patients (92%) with LM from EGFR-mutated NSCLC [92]. However, some technical issues must be considered: most of the studies used digital PCR or targeted sequencing of a limited number of genes and did not cover the whole range of the targetable mutations. Moreover, copy-number mutations and some gene translocations and fusions, such as ALK-rearrangement, may be not detected using the standard “off the shelf” whole-exome sequencing. In addition, most clinical institutions do not have the professional and technical resources to perform the analyses “in house”; thus, samples must be sent to other adequately equipped facilities, and issues of sample managing, storage and shipment must be addressed [93].

Unfortunately, there is a paucity of studies comparing the accuracy of CTCs and ctDNA in both blood and CSF of patients with LM. It is unknown whether CTCs or ctDNA are superior for defining the genetic profile, as well as the spatial and temporal heterogeneity of tumors. However, the combination of CTCs and ctDNA may provide comprehensive information in terms of heterogeneity of tumor cells and prognosis. In this regard, Nevel et al. reported that patients with ≥50 CTCs/3 mL had an increased risk of death in comparison with that of those with <50 CTCs/3 mL, as well as that increased ctDNA concentrations were correlated with an increased risk of death [75]. Major concerns arise from the evidence that not all ctDNA may be tumor derived, and it could be too early to state that increased ctDNA concentrations in CSF could predict OS. The mutational status of LM may also impact survival. Zheng et al. reported that intracranial progression-free survival (iPFS) in patients with LM from NSCLC was higher in those harboring the EGFR exon 19 deletion (11.9 months) than in those with EGFR exon 21 L858 mutation (2.8 months). Moreover, the median iPFS was longer in patients with EGFR T790-positive CSF genotyping (15.6 months) than in those without T790 mutation (7.0 months). The concomitant presence of CD42 (2.8 months) and CDKN2A mutations (2.5 months) confers a shorter iPFS (11.6 and 9.6 months, respectively) than that of patients with negative CSF-ctDNA. Lastly, some resistant mutations, such as EGFR C795 mutation, MET dysregulation, co-occurrence of TP53 and RB1 mutations and loss of T790 mutation in CSF-ctDNA, were correlated with shorter survival in patients who progressed with LM after treatment with osimertinib [97]. Overall, the integration of the CSF liquid biopsy in the diagnostic flowchart may lead to several advantages in the management of LM, including diagnosis of LM in case of negative or “atypical” CSF cytology, monitoring of tumor response following targeted therapy or immunotherapy, early detection of LM recurrence and development of resistant mutations, early identification of subgroups of patients with a higher risk of LM recurrence and correlation of LM burden with survival. However, these attractive goals may only be achieved after collection and analysis of larger datasets of CSF liquid biopsy; thus, the rarity of LM requires a cooperative approach to make CTC and ctDNA data available to a larger community of basic and clinical researchers.

A new frontier in CSF liquid biopsy is proteomics. Smalley et al. collected 45 consecutive CSF samples from 16 patients with LM from melanoma: CSF was analyzed by mass spectrometry and incubated with melanoma cells, and RNA sequencing was performed. The mass spectrometry analysis revealed that the CSF of most LM was significantly enriched for pathways involved in innate immunity, protease and IGF-mediated signaling. Furthermore, RNA sequencing showed a significant activation of the PI3K/AKT pathway, integrin, TNFR2 and TGF-β, as well as B-cell activation and oxidative stress that were correlated with leptomeningeal progression, development of resistance to BRAF inhibitors and poor survival [101].

## 5. Targeting LM with Systemic and Intrathecal Approaches

**Figure 4:**
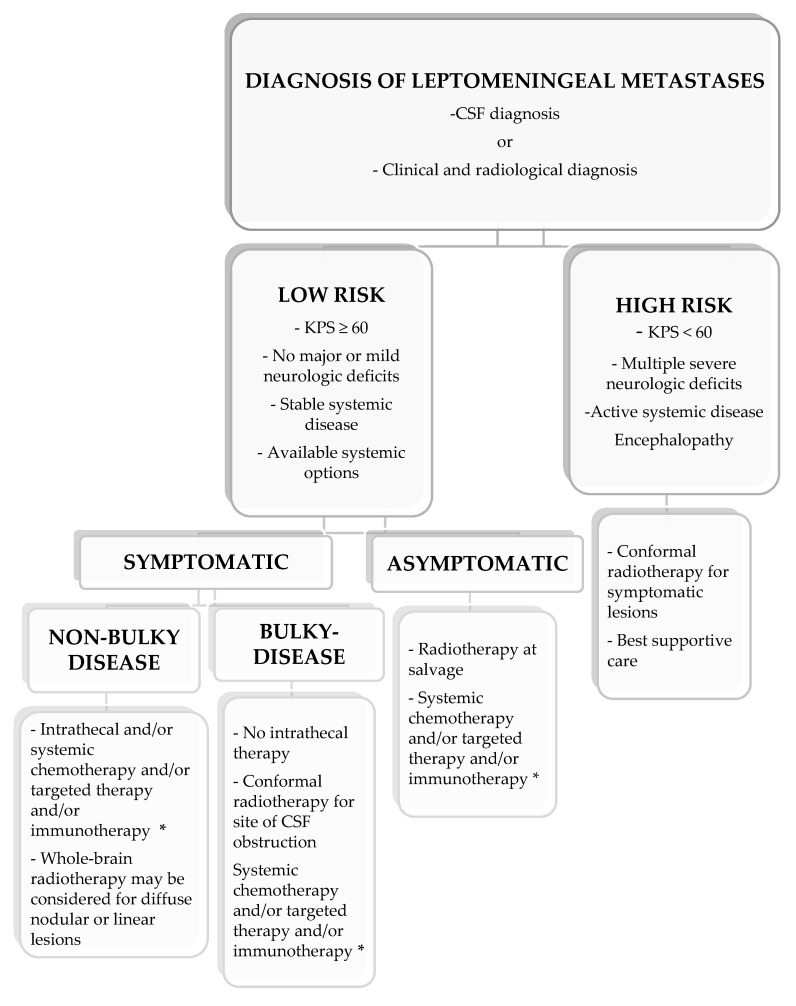
Treatment decision algorithm for leptomeningeal metastases. * Based on druggable mutations or PD-L1 expression.

Surgery may be useful to relieve increased intracranial pressure with the placement of ventriculo-peritoneal shunts. Ommaya reservoirs may also serve for the administration of intrathecal therapies. Different techniques of radiotherapy (RT) may be considered for the treatment of LM. In this regard, focal RT, such as involved field or SRS, may be delivered in selected patients with circumscribed and symptomatic lesions or with CSF flow obstructions for palliation or improvement of the distribution of intrathecal drugs. Whole-brain radiotherapy (WBRT) has been investigated in unfit patients with poor performance status in LM from different solid tumors and reported a modest benefit in neurological symptoms and pain control [102,103]. More aggressive approaches with craniospinal irradiation (CSI), using either conventional RT [104,105] or proton therapy [106], have been investigated with the aim to control the disease of the whole neuroaxis, but limited data on efficacy are available thus far. Overall, CSI is not typically considered the standard of care. Targeted therapy, immunotherapy and intrathecal therapy in selected patients may represent an optimal treatment for LM according to the molecular subtypes (Figure 4)**.**

### 5.1. LM from EGFR-Mutated NSCLC

LM occur more frequently in EGFR-mutated (9.4%) compared with EGFR wild-type NSCLC (1.7%) with a median OS of 13.3 months [107,108]. The first-generation TKIs have limited penetration into the CSF (1–3%). The intensification of gefitinib or erlotinib using a “pulsatile” regimen has been proposed to increase the CSF concentrations with a median OS ranging from 3.5 to 12 months [109,110,111,112,113]. The second-generation TKI afatinib was evaluated in a prospective trial on 11 patients with LM pretreated with first-generation TKI. Five patients harbored an exon 19 deletion, three harbored a p.L858R point mutation, and three harbored an uncommon exon 18 mutation. A radiological response was achieved in 27.3% of patients, of whom two out of three harbored uncommon EGFR mutations, with a median PFS of 2.0 months and OS of 3.8 months [114]. A CNS recurrence following first- and second-generation TKIs occurs in 40% of patients due to the limited ability to cross the BBB and the development of resistant mechanisms. In this regard, the third-generation TKI osimertinib has shown a higher penetration through the BBB (CSF level 7.51 and 25.2 nmol/L when administered at 160 mg/day and 300 mg bid, respectively) and now is considered the first-line therapy in LM from EGFR-mutated NSCLC based on the results of several studies [115,116,117,118,119,120,121,122,123], regardless of T790 mutation status [121] (Table 4). The studies reported an intracranial response rate of 20–62%, a median PFS of 7.2–17.2 months, a median OS of 11–18 months and a rapid neurological improvement in the majority of patients [118], as well as a clearance of CSF from neoplastic cells in 28% [117]. Interestingly, osimertinib also displayed significant activity in patients harboring uncommon EGFR aberrations, including Leu858Arg [116] and S768I [122], and 750_758del, I759S and T751_I759delinsS mutations [123]. A preplanned analysis of the phase 3 FLAURA study investigated osimertinib in comparison with first-generation TKIs as un upfront treatment in metastatic Exon19 deleted/L858R EGFR-mutated NSCLC, showed that four out of five patients had a complete radiological response of LM. Together, these data suggest that osimertinib should be considered as the preferred initial treatment when feasible [124]. As pemetrexed has displayed some activity to control LM [125], the FLAURA2 trial is now investigating the efficacy of the association of osimertinib and pemetrexed in both BM and LM (NCT04035486) [126]. Another treatment option under investigation is the combination of bevacizumab and TKIs. A case report of LM from NSCLC treated with erlotinib and bevacizumab reported a neurological improvement and stabilization of disease lasting 18 months [127]. Osimertinib was investigated also in association with bevacizumab in a patient with LM displaying a durable clinical and radiological response of 10 months [128], and an ongoing trial is now addressing this combined treatment in LM (NCT04425681, NCT04148898) (Table 5).

AZD3759 is a novel compound primarily designed to cross the BBB that has displayed a remarkable ability to penetrate into the CSF, as well as significant efficacy in three out of four patients with LM in a phase I trial [130]. Cho et al. reported a radiological response in 5/18 patients (27.8%) and a stable disease in 9/18 (50%) following two different doses of AZD3759 (200 and 300 mg, respectively) without significant difference in tolerability [131]. A newer generation of TKIs have been investigated: nimotuzumab led to a significant radiological response in two out of three LM [132], while tesevatinib showed activity in disease control of advanced NSCLC, and a clinical trial on BM and LM has been completed in January 2020, and the results are awaited (NCT02616393).

### 5.2. LM from ALK-Rearranged NSCLC

ALK mutations are rare and can be found in approximately 3–7% of patients with NSCLC. ALK-rearranged NSCLC recurs in approximately in 35–40% of patients with BM and in 5% of patients with LM after a median time of 9 months from the diagnosis of the primary tumor [143].

ALK inhibitors have changed the natural history and prognosis of advanced NSCLC, including patients with BM, but data on LM are limited to case reports. Although the first-generation ALK inhibitor crizotinib has a poor penetration through the BBB (CSF level 0.14 nmol/L when administered at 250 mg/day), some cases have been reported with a prolonged PFS (6–10 months) when crizotinib is given following WBRT or concurrent with intrathecal methotrexate (MTX) in LM [133,134]. The second-generation ALK inhibitor ceritinib displayed some activity (PFS 5–18 months) in LM when combined with traditional chemotherapy or WBRT after failure of crizotinib [135,136]. Three different ASCEND trials have shown a significant intracranial response rate following ceritinib in patients with BM, heavily pretreated with chemotherapy and crizotinib; however, no details regarding LM response have been reported [144,145,146]. Alectinib is a second-generation ALK inhibitor with a higher CSF penetration (2.69 nmol/L) that became the preferred first-line therapy in ALK-rearranged patients according to the phase 3 ALEX study, where alectinib was compared with crizotinib [147]. To date, a total of six patients with LM received alectinib with a daily dose ranging from 600 to 900 mg, reporting a durable neurological and radiological improvement and a median PFS of 3.5–15 months [137,138,139]. The third-generation ALK-inhibitors brigatinib and lorlatinib have displayed significant intracranial activity compared to the older generations of ALK-inhibitors [148,149,150,151]; however, the activity on LM has not been fully investigated. To date, two different case reports displayed a prolonged PFS in LM following brigatinib and lorlatinib, respectively [140,141]. In an interim analysis of the results from the CROWN study among patients with BM, those who received lorlatinib achieved an intracranial objective response rate (iORR) of 82%, while those treated with crizotinib had an iORR of only 23%. Notably, 71% of the patients who received lorlatinib had an intracranial complete response, suggesting major intracranial activity of lorlatinib compared with other ALK-TKIs [151]. Recently, Frost et al. reported the results of the German early access program on lorlatinib in 36 patients with symptomatic BM and 9 LM after the failure of first- and second-generation ALK inhibitors: an intracranial response rate of 54%, a median duration of treatment of 10.4 months and a median PFS of 8.0 months were reported. Overall, this is the first real-life experience showing the efficacy of lorlatinib in heavily pretreated patients with BM and LM and also in patients harboring resistance mutations (e.g., G1202R and G2032R mutations) [142].

### 5.3. LM from Breast Cancer

BC expresses different molecular markers, including estrogen receptors (ER), progesterone receptors (PR) and HER2, leading to molecular subtypes with a different risk of developing CNS recurrences and survival; thus, the molecular profile should be obtained to tailor treatments [152]. In fact, patients with triple-negative BC (TNBC) show the highest incidence of LM (36%), with a shorter time to development of LM and OS [153,154], while LM occur approximately in 14% of patients with ER/PR-negative and HER2-positive BC and in 2.2% of patients with luminal A (low-grade and ER-positive) BC [155].

#### 5.3.1. HER2-Positive LM from Breast Cancer

Trastuzumab has been demonstrated to prolong OS (15.2 versus 9.9 months) and delay the onset of BM in HER2-positive BC, suggesting a preventive role by blocking the entry of tumor cells into the CNS [156]; however, CNS is the first site of relapse following trastuzumab due to the poor ability to cross the BBB [11,157,158]. Therefore, the efficacy of trastuzumab was evaluated when administered intrathecally and compared with intrathecal MTX/thioTEPA or WBRT: prolonged LM control of more than 10 months in four patients treated with intrathecal trastuzumab was reported, as well as a one-year OS of 54% compared with 10% following intrathecal MTX/thioTEPA and 19% following WBRT [159]. Recently, Zagouri et al. conducted a meta-analysis on intrathecal trastuzumab in patients with LM from HER2-positive BC, reporting a median PFS and OS of 5.2 and 13.2 months, respectively, as well as a CSF clearance in 56% of patients and a radiological improvement or stabilization in nearly 71% [160]. As some old studies reported a limited palliative activity of standard intrathecal chemotherapy (MTX, liposomal ara-C and ThioTEPA) [161,162,163,164,165], intrathecal trastuzumab may be more effective in terms of LM control and outcome when compared with historical cohorts, but further prospective trials are needed. The association of trastuzumab, pertuzumab and docetaxel is considered the standard first-line treatment in HER2-enriched advanced BC, according to the CLEOPATRA trial; however, data on activity in BM or LM are lacking, as the enrollment of patients with CNS recurrences were not allowed [166]. A clinical trial of intrathecal pertuzumab/trastuzumab in association with focal RT or WBRT in LM is underway (NCT04588545) (Table 5).

The antibody-drug conjugate trastuzumab emtansine (TDM-1) represents a further advancement to treat metastatic HER2-positive BC with significant activity in both asymptomatic and symptomatic BM [167,168,169], but little is known regarding its efficacy in LM. To date, one case report reported a clinical and radiological response lasting >3 months after the association of TDM-1 with WBRT in a patient with HER2-positive LM [170]. The evolution of TDM-1 is represented by trastuzumab deruxtecan, which is now under investigation in a distinct cohort focused on HER2-enriched LM of the DEBBRAH trial (NCT04420598) (Table 5).

New HER2-TKIs, such as lapatinib, neratinib and, in particular, tucatinib, have improved better penetration into the CNS and displayed an impact in intracranial disease control in BM of patients pretreated with trastuzumab, especially when administered with capecitabine [171,172,173]. Freedman et al. enrolled three patients with LM pretreated with lapatinib and reported one partial response after seven cycles of capecitabine plus neratinib and one stable disease and one progressive disease after four cycles, respectively [171]. An Italian cohort of heavily pretreated LM (median number of adjuvant therapies of 3) received capecitabine plus neratinib as part of a compassionate program: the median PFS and OS were 4.0 and 10 months, respectively. Moreover, a neurological improvement was reported in two out of seven patients (28.6%), while in three out of seven patients (42.8%), a neurological stabilization was achieved, lasting for a median time of 5 months. The best radiological response was stable disease in four out of seven patients (57.1%), while no complete or partial responses were achieved [174]. To date, no data are available regarding the activity of tucatinib in LM: a phase 2 trial has already enrolled 30 patients with LM who received trastuzumab plus capecitabine and tucatinib (NCT03501979) (Table 5).

In a separate study of non-HER2-directed therapy, Lu et al. reported an intracranial response in 19/34 HER2-positive patients (68%) affected by LM who received bevacizumab in combination with etoposide and cisplatin (BEEP regimen), with a median OS of 13.6 months [175].

#### 5.3.2. ER-Positive LM from Breast Cancer

A small number of case reports have described a benefit from hormonal therapy (HT), which consists of tamoxifen or fulvestrant in pre-perimenopausal women, or a luteinizing hormone releasing hormone (LHRH) agonist for post-menopausal women [176,177,178,179]. The introduction in clinical practice of CDK4/6 inhibitors, which inhibit cyclin D1 pathways and arrest the proliferation of ER-positive BC cells, in combination with estrogen therapy or an LHRH agonist, had a minor impact on intracranial disease control in BM [180,181,182], but the activity in LM has not been investigated thus far. New treatment strategies are urgently needed: in this regard, the estrogen receptor degraders (SERDs) aim to block the ER pathway, and some clinical trials on metastatic BC are ongoing (NCT02248090, NCT2338349).

#### 5.3.3. LM from Triple-Negative BC

The standard of care for advanced TNBC is represented by platinum-based chemotherapy, such as carboplatin, which has demonstrated better tolerability compared with docetaxel, regardless of BRCA (breast cancer susceptibility genes 1 or 2) status [152]. The mutations of BRCA1/2 genes impair the ability of the poly adenosine diphosphate ribose polymerase (PARP) enzymes to repair the DNA double-strand breaks that lead to the apoptosis of tumor cells. Different PARP inhibitors, including iniparib, olaparib, talazoparib and veliparib, demonstrated some activity in metastatic TNBC, including in patients with asymptomatic BM [183,184]. To date, two case reports have displayed some activity of olaparib in LM from BRCA1/2 mutated TNBC. Bengham et al. described a dramatic response of LM of the spinal cord and the skull base after 4 months from the start of olaparib, with a duration of 12 months. Clinical and radiological improvement was also confirmed by the clearance of CSF from neoplastic cells, as well as a reduction of the CSF level of CA-125 [185]. A similar result was reported by Exman et al. in a patient with BRCA2 mutation achieving a complete neurological and radiological response following olaparib after 19 months of therapy [186]. Further investigations are warranted allowing the enrollment of patients with LM in trials that aim to address the efficacy of PARP inhibitors in TNBC harboring BRCA1/2 aberrations.

When druggable mutations are not expressed on tumor cells, immunotherapy may represent an option in TNBC. However, a recent trial on atezolizumab on advanced and metastatic TNBC did not show a significant benefit in patients with BM [187]. Despite these results, several clinical trials are now evaluating the role of immune checkpoint inhibitors (ICIs), such as nivolumab (NCT03807765), pembrolizumab (NCT03449238) and atezolizumab (NCT03483012), in combination with SRS in BM from TNBC. Recently, a single-arm, phase 2 study of pembrolizumab in 20 patients with LM from solid tumors (17 BC, 2 NSCLC and 1 ovarian cancer) showed promising results. Twelve out of twenty patients (60%) met the primary endpoint of three-month OS (60%) with a manageable toxicity (40% of grade 3 adverse events). Further analyses are ongoing to identify subgroups of patients that may benefit from anti PDL-1 treatment [188].

Modifying the structure of traditional chemotherapy and linking it to peptide vector or pegylation is another strategy to increase the penetration through the BBB. The taxane agent ANG1005 consists of three paclitaxel molecules covalently linked to angiopep-2, which is able to cross the BBB by the LRP1 transport system. Kumthekar et al. conducted a phase II study on intravenously ANG1005 in 72 patients with CNS recurrences from BC, including 28 with LM, and reported a clinical benefit in 77% of patients and an intracranial response rate in 15%. Of note, 79% of patients with LM had disease control with a median OS of 8.0 months [189]. These encouraging results have led to other randomized trials to validate this compound in recurrent BM (NCT02048059) and LM (NCT03613181).

### 5.4. LM from Melanoma

About 50% of advanced melanoma has mutations in position 600 (v600) of the serine/threonine kinase BRAF with some evidence of increased risk of progression in the CNS [190]. Although targeted therapy can reach adequate levels in the CSF, there is wide interpatient variability of vemurafenib concentrations, reflecting the different permeability of BTB. The combination of local therapy, including surgery and RT, can impact the BTB permeability with the highest levels of vemurafenib achieved following SRS [191]. Few case reports reported a clinical and radiological response after BRAF inhibitors or MEK inhibitors, with prompt neurological symptom relief and CSF cytological remission [189,192,193,194,195,196,197]. Arasaratnam et al. reported an advantage of BRAF inhibitors in a cohort of 11 patients with LM from melanoma [190]. Interestingly, patients who continue to receive BRAF inhibitors beyond progression, as well as patients who received treatment at the time of diagnosis of LM, had benefited from BRAF inhibitors with a median OS of 7.2 months. It is not clear whether the association of BRAF inhibitors and MEK inhibitors may improve the efficacy in LM as reported in BM and extracranial sites [198]; however, initial reports regarding the combination of targeted therapy and immunotherapy with RT are emerging. In this regard, 28 patients with LM were treated with targeted therapy (*n* = 5), traditional chemotherapy (*n* = 1), anti-PD-1 alone (*n* = 17) or in combination with a BRAF inhibitor (*n* = 4), achieving a median OS of 7.1 months for the patients receiving systemic therapy combined with RT and 3.2 months for those not receiving RT [199].

Immune checkpoint inhibitors (ICIs) have drastically changed the natural history and survival of metastatic melanoma patients, including those with BM, while the knowledge regarding the activity on LM mainly derives from few case reports, using WBRT in combination with ipilimumab [200] or anti-PD-1 [197], with durable neurological improvement. Cohort C of the ABC phase II study enrolled four patients with LM who received nivolumab as a single agent, but none of them responded, achieving a poor median OS of 5.1 months [201]. Intrathecal immunotherapy has also been considered in LM from melanoma, with some concerns regarding the possibility of inducing dramatic inflammation in the CNS and, though rare, severe neurological adverse events. The cytokine interleukin (IL)-2 was delivered intrathecally in a cohort of patients with LM from melanoma: a median OS of 9.1 months was achieved with one-year, two-year and five-year OS of 36, 26 and 13%, respectively. However, severe adverse events were reported, including increased intracranial pressure, that required an intensive care observation [202]. When intrathecal IL-2 therapy fails, other innovative strategies have been investigated, such as educated cytotoxic T lymphocytes (cyt-T cells) after the interaction with autologous dendritic cells charged with different melanoma antigens (melanoma-associated antigens tyrosinase, Melan-A/MART, gp100/Pmel17). One patient only received cyt-T cells by Ommaya reservoir showing increased CSF TNF-α, IFN-γ and IL-6 concentrations as well as an OS > 18 months from the diagnosis of LM [203]. One other patient received cyt-T cells intrathecally after the failure of IL-2: the patient died after 5 months from the start of treatment for systemic progressive disease, but LM remained stable [204]. The safety of nivolumab was investigated intrathecally via an intraventricular reservoir in association with an intravenous route in 15 patients with evidence of LM on MRI and/or CSF cytology from heavily pretreated metastatic melanoma (anti-PD-1: 11 patients; BRAF/MEK inhibitors: 9 patients; traditional chemotherapy: 2 patients; intrathecal IL-2: 4 patients; other therapies: 2 patients). Two patients received intrathecal nivolumab at 5 mg, 3 patients at 10 mg and 10 patients at 20 mg. No grade 4–5 adverse events were reported with intrathecal or intravenous nivolumab. With a median follow-up of 18.7 weeks, the median OS was of 46.1 weeks (0.1–83.3). Clinical response data and translational research endpoints, including changes in CSF cytokines and cfDNA, are still under investigation [205].

## 6. Novel Techniques to Improve Drug Delivery across the BBB

Novel techniques are under investigation to overcome the limits posed by BBB and improve the penetration of drugs into the CNS. In this regard, cranial implantable ultrasound emitters, when combined with microbubbles intravenously, transiently disrupts the BBB, and the penetration of larger and polar molecules into the CNS is more feasible. The BBB opening is reversible and lasts several hours after the ultrasound application. However, the thick human skull represents a barrier for the penetration of ultrasounds; thus, the implanting of an ultrasound emitter into a window on the patients’ skull is mandatory to overcome the intrinsic resistance of the skull [206] and could result in further discomfort for patients with symptomatic LM. Idbaih et al. have reported the successful opening of the BBB in 52 of 65 sonication sessions in 19 patients with recurrent glioblastomas using an implantable ultrasound emitter in combination with carboplatin [207]. Several clinical trials are now evaluating the ability to open the BBB by cranial ultrasound emitters, but they are reserved for circumscribed high-grade gliomas or brain metastases and not for a diffuse disease with multilevel involvement of the neuroaxis, such as LM. Moreover, the impact of ultrasounds to disrupt the BTB and the CSF barriers, as well as the ability to improve the CSF concentrations of targeted therapy and/or immunotherapy in LM, is unknown. An additional problem is the rapid turnover of CSF, which leads to fluctuating concentrations and shorter half-life of compounds in the CSF, as well as a limited exposure of floating tumor cells to antineoplastic agents. Gene therapy has been suggested to solve the problem of larger compound delivery through the BBB in one shot administration. Adeno-associated viral (AAV) vectors, particularly serotype 9, can deliver exogenous genes, such as the gene for trastuzumab, to the entire neuroaxis after a single intrathecal administration, leading to a durable and stable expression of the transgene product in both CNS and CSF. Rothwell et al. reported in an orthotopic Rag1-/- murine xenograft model of HER2-positive BM from BC that a single prophylactic intrathecal administration of an AAV9-trastuzumab vector increased the median OS (124 versus 50 days), attenuated brain tumor growth and preserved both the HER2 antigen specificity and the natural killer cell–associated mechanism of action of trastuzumab. The authors stated that they intend to move AAV9-trastuzumab toward a human clinical trial after the completion of preclinical studies, including safety and toxicology experiments in large animal models. The next step will be to assess the safety, efficacy and pharmacokinetics of AAV9-trastuzumab in women with documented CNS lesions from HER2-enriched BC. Of note, as AAV transgene expression has been reported to persist for years in primates and humans, this approach has been suggested as a potential additional part of the adjuvant therapy with the aim to prevent CNS recurrences for patients with early diagnosis of HER2-positive BC [208].

## 7. Conclusions

Leptomeningeal space remains a sanctuary site, and little is known about the microenvironment of LM. The peculiar adaption to compartments with different metabolic features, such as the brain parenchyma and circulating CSF, selects unique intracellular survival pathways during proliferation and clonal selection, leading to growth of tumor cells in two distinct anatomical compartments [209]. Therefore, the mechanisms underlying the invasion of the CNS as well as the interaction of tumor cells with either the brain parenchyma or leptomeningeal space may be regulated by distinct pathways based on molecular subgroups that need to be further investigated. Overall, targeted therapy and immunotherapy may be active on cells in specific contexts, but a better understanding of molecular pathways that regulate the penetration of different compounds through the BBB/BTB interface is limited.

The current knowledge of efficacy of newer intrathecal and targeted or immunotherapy approaches primarily derives from case reports or analyses of small subgroups of patients in clinical trials. Thus far, these new treatment options impact a small percentage of patients with LM. An urgent need is to design clinical trials on LM for longitudinal CSF, blood and tissue collection at diagnosis and during treatment to monitor clinical and radiological response, obtain pharmacokinetic information and identify markers of response and resistance. In this regard, different combinations of treatments, such as EGFR TKIs with antiangiogenic agents and intrathecal chemotherapy or antiangiogenic therapy with anti-PD-L1 in LM from NSLCLC, as well as intrathecal chemotherapy or ant-PD-L1 with RT in LM from BC, are under investigation in clinical trials (Table 5). However, due to the rarity of LM, a multicenter cooperative effort is crucial to achieve a significant sample size in order to derive robust results regarding the efficacy of treatments.

## Figures and Tables

**Table 1:** Blood–brain barrier/blood–tumor barrier heterogeneity in preclinical and clinical studies.

Preclinical Models
**NSCLC**
BM models using the ALK-rearranged NSCLC cell line H3122 EML4-ALK^L1196M^ showed that the PF-06463922 compound is a potent brain-permeable ALK/ROS1 inhibitor with an increased ability to cross the BBB/BTB and penetrate into non-permeable micrometastases and macrometastases [22]
**Melanoma**
An in vivo longitudinal MRI study using contrast-enhanced T1-weighted spin echo images was performed after the intracardiac injection of melanoma cell lines A2058 in mice, reporting the presence in BM of regions with intact BBB mixed with other areas with a disrupted BBB [23]
BM models from melanoma cells (MDA-MB-435 and A2058) show vessel cooperation between tumor cells and BTB, while models using NSCLC cells (PC14-PE6 and HTB177) enhance the neoangiogenesis to alter the BBB/BTB [24]
**Breast Cancer (BC)**
After the intracardiac injection of BC cell lines 231-BR-HER2 and 4T1-BRS, the BTB permeability differs between the two models: the 231-BR model showed a significant permeability to drugs because of the high expression of S1PR-3 [19]
The BBB is more permeable in cell models using SUM190-BR3 and JIMT-1-BR3 BC cells compared with the 231-BR-HER2 counterpart [25]
Desmin-positive pericytes correlate with areas of high permeability on BTB from BC [17]
Intracranial injection model using human BC cell line BT474 is permeable to chemotherapy and antibodies in physiological conditions, but the drug delivery significantly increases after the BBB/BTB disruption [26]. Such a preclinical model has been reproduced in a clinical setting demonstrating a comparable drug delivery of targeted therapy (lapatinib) in BM and primary site [27]. Furthermore, intracranial injection of BT474 cells creates a leakier BBB/BTB and an increased drug uptake compared with intravenously or intracardially injection models, displaying that the route of administration significantly impacts the drug delivery [28]
**Miscellanea**
Some patient-derived xenograft models of CNS recurrences from NSCLC, BC, melanoma, prostate and neuroendocrine tumors revealed a decreased expression of NLS1 on BBB compared with healthy brain tissue [18]
**Clinical Data**
**Breast Cancer**
BBB/BTB interface is different according to molecular subtypes of BC. HER2-enriched cells tend to preserve the integrity of the BBB, while TNBC or basal-type BC disrupt the BBB. Moreover, the ECs of BTB express higher levels of GLUT1 and BCRP compared with those ER-positive or TNBC [20]
Lapatinib achieves significant concentrations (1.0–6.5 microM) from HER2-positive BM when administered at a daily dose of 1250 mg (last dose 2–3 h before surgery) [29]. This evidence was confirmed by PET studies when comparing tumors with normal brain tissue [30]. Although HER2-positive BM preserve the integrity of BBB, trastuzumab alone or linked to emtansine (TDM-1) achieves a significant distribution due to the leakiness of the BTB [31,32]

NSCLC: non-small-cell lung cancer; BM: brain metastasis; ALK: anaplastic lymphoma kinase; BBB: blood–brain barrier; BTB: blood–tumor barrier; MRI: magnetic resonance imaging; CNS: central nervous system; NLS1: sodium-dependent lysophosphatidylcholine symporter 1; TNBC: triple-negative breast cancer; ECs: endothelial cells; GLUT1: glucose transporter 1; BCRP: breast cancer resistance protein; ER: estrogen receptor; PET: positron emission tomography.

**Table 2:** Studies on circulating tumor cells in CSF from LM.

Study	Number of Patients	Type of Primary Solid Tumor	Results
Patel et al., 2011 [74]	5	BC	CTCs detected using CellSearch technique showing that the number of CTCs are correlated with leptomeningeal burden and response to chemotherapy
LeRhun et al., 2012 [80]	8	BC	Detection of CTCs with adapted CellSearch technology displays a quantitative correlation with the response to therapy
Subirà et al., 2012 [63]	72	BC (44) NSCLC (23) GIC (4) Cavum (1)	Sensitivity of CTCs: 75.5% Sensitivity of CSF cytology: 65.3% Specificity of CTCs: 96.1% Specificity of CSF cytology: 100% Immunoflow cytometry
Nayak et al., 2013 [64]	51	NSCLC (21) BC (15) Melanoma (1) Ovarian cancer (2) Others (12)	Sensitivity of CTCs: 100% Sensitivity of CSF cytology: 66.7% Specificity of CTCs: 97.2% Specificity of CSF cytology: 100% Median CTCs: 20.7 cells/mL CellSearch technique
LeRhun et al., 2013 [72]	2	Melanoma	First study that used adapted CellSearch technology against HMW-MAA to detect melanoma CTCs
Lee et al., 2015 [66]	38	BC	Sensitivity of CTCs: 80.9% Sensitivity of CSF cytology: 66.7% Specificity of CTCs: 84.6% Specificity of CSF cytology: 100% CellSearch technique
Subirà et al., 2015 [65]	144	BC (39) NSCLC (35) GIC (6) Ovarian (4) Prostate (3) Others (5)	Sensitivity of CTCs: 79.8% Sensitivity of CSF cytology: 50.0% Specificity of CTCs: 84.0% Specificity of CSF cytology: 100% Immunoflow cytometry
Tu et al., 2015 [67]	18	NSCLC	Sensitivity of CTCs: 77.8% Sensitivity of CSF cytology: 44.4% Specificity of CTCs: 100% Specificity of CSF cytology: not reported CellSearch technique
Acosta et al., 2016 [81]	6	Epithelial cell tumors	Sensitivity of CTCs: 100.0% Specificity of CTCs: 100.0% Immunoflow cytometry
Milojkovic Kerklaan et al., 2016 [68]	29	Epithelial cell tumors	Sensitivity of CTCs: 100.0% Sensitivity of CSF cytology: 61.5% Specificity of CTCs: 100.0% Specificity of CSF cytology: 100% Immunoflow cytometry
Ma et al., 2016 [82]	10	NSCLC	Seven out of ten CSF samples where CTCs were found with a range from 3 to 1823 tumor cells TM-iFISH
Jiang et al., 2017 [76]	21	NSCLC	Sensitivity of CTCs: 95.2% Sensitivity of CSF cytology: 57.1% Specificity of CTCs: 100% Specificity of CSF cytology: not reported CellSearch technique
Lin et al., 2017 [83]	95	NSCLC (36) BC (31) Others (28)	Sensitivity of CTCs: 93.0% Sensitivity of CSF cytology: 29.0% Specificity of CTCs: 95.0% Specificity of CSF cytology: not reported CellSearch technique
van Bussel et al., 2020 [84]	81	NSCLC	Sensitivity of CTCs: 94.0% Specificity of CTCs: 100.0% Cut-off for CTCs positivity: 0.9 CTC/mL Immunoflow cytometry
Nevel et al., 2020 [75]	16	NSCLC	Patients with ≥50 CTCs/3 mL had an increased risk of death in comparison with that of those with <CTCs/3 mL CellSearch technique
Malani et al., 2020 [85]	15	HER2-positive BC	CSF CTCs were identified in 13 patients (87%) Median CSF CTCs was 22 CTCs/3 mL (range 0–200 +). HER2 expression analysis of CTCs was performed in 8 patients; 75% had confirmed expression of HER2 in CSF CellSearch technique

BC: breast cancer; CTCs: circulating tumor cells: NSCLC: non-small-cell lung cancer; GIC: gastrointestinal cancer: CSF: cerebrospinal fluid; HMW-MAA: human high molecular weight-melanoma-associated antigen; TM-iFISH: tumor marker-immunostaining fluorescence in situ hybridization; HER2: human epidermal growth factor receptor 2.

**Table 3:** Studies on circulating tumor DNA in CSF from LM.

Study	Number of Patients	Type of Primary Solid Tumor	Results
Swinkels et al., 2000 [90]	2	NSCLC	KRAS mutation was found in CSF of 2/2 patients (100%) CSF Mutant-allele-specific amplification (PCR)
Momtaz et al., 2016 [87]	11	BRAF-mutated malignancies	BRAF mutations detected in CSF-ctDNA of 6/11 patients (54%) Droplet digital sequencing
Pentsova et al., 2016 [88]	32 BM 9 LM	NSCLC (11) BC (11) Melanoma (6) Others (13)	Mutations were detected in CSF-ctDNA of 20/32 patients (63%) with BM and 3/4 patients (75%) with LM Targeted sequencing
Marchio et al., 2017 [89]	2	NSCLC	KRAS mutations detectable in CSF-ctDNA of 2/2 patients (100%) Targeted sequencing
Fan et al., 2018 [91]	11	EGFR-mutated NSCLC	EGFR mutations were found in CSF-ctDNA of 11/11 patients (100%). Mutations were not concordant in 1/11 (9%) Targeted sequencing
Li et al., 2018 [92]	42	EGFR-mutated NSCLC	Distinct EGFR mutations were found in CSF-ctDNA of 28 patients (92%) Targeted sequencing
Huang et al., 2019 [94]	20 BM 15 LM	EGFR-mutated NSCLC	EGFR mutations were detected in 23/35 patients: BM: blood: 6/11 (54.5%); CSF: 5/10 (50%) LM: blood: 4/11 (36.4%); CSF: 9/12 (75%) T790 mutation was significantly higher in blood (9/23) than that of CSF (3/23) Sensitivity in CSF: 56%; in blood: 89% Specificity in CSF: 46%; in blood: 100% Twelve patients received a first-generation TKI after the detection of actionable mutation in CSF, while 5 patients switched to osimertinib after the detection of T790 mutation in CSF or blood Droplet digital PCR
Ma et al., 2020 [95]	11	NSCLC that progressed after 3rd generation TKIs	CSF-ctDNA was identified in 8/11 patients (72.7%) and in the blood of 6/11 (54.5%) EGFR C797 mutation and MET amplification were found in CSF of 4/11 patients (36.3%) and in the blood of 2/11 (18.2%) One patient only had C797 and T790 mutation concurrently Longitudinal assessment with CSF-ctDNA displayed that the level of C797 mutation decreased with radiological and neurological improvement, while the blood level of T790 mutation increased early before leptomeningeal progression Nanowire-based ctDNA assay
Li et al., 2020 [96]	18	NSCLC EGFR-mutated (11) ALK-rearranged (6) ROS1-mutated (1)	The MET mutational rate was higher in CSF (100%) than that of blood (66.7%) A higher number of SNVs and copy number variants were found in CSF in comparison with blood SNVs were higher in patients pretreated with ≥2 TKIs than that of those who received 1 TKI only NGS
Nevel et al., 2020 [75]	21	NSCLC	CSF-ctDNA concentrations ranged from 0.093 pg/microL to 0.562 ng/microL Median CSF-ctDNA concentration was 0.022 ng/microL An increased risk of death was observed when ctDNA concentrations were higher than the median cutpoint Targeted exome sequencing MSKCC IMPACT
Zheng et al., 2021 [97]	80	EGFR-mutated NSCLC Cohort 1: CSF and blood genotyping before the 1st administration of osimertinib (45) Cohort 2: CSF genotyping at the time of progression with LM during treatment with osimertinib (35)	Detection of actionable EGFR mutations in CSF-ctDNA: Cohort 1: 42/45 (93.3%) Cohort 2: 34/35 (97.1%) Median iPFS was higher in patients with EGFR exon 19 deletion (11.9 months) compared with that of patients harboring EGFR exon 21 L858 mutation (2.8 months) Median iPFS was higher in patients with EGFR T790-positive CSF genotyping (15.6 months) than that of those without T790 mutation (7.0 months) Concomitant presence of CD42 (2.8 months) and CDKN2a mutations (2.5 months) confers a shorter iPFS (11.6 and 9.6 months, respectively) than that of patients with CSF-ctDNA negative Cohort 2: EGFR C795 mutation, MET dysregulation, co-occurrence of TP53 and RB1 mutations as well as loss of T790 mutation in CSF-ctDNA were correlated with shorter survival NGS
Carausu et al., 2019 [98]	1	HR-positive/HER2-negative BC	First report of detection of CSF-ctDNA of ESR1 mutation after treatment with aromatase inhibitor Droplet digital PCR
Angust et al., 2021 [99]	151	BC	Thirty CSF samples were analyzed with NGS and 121 with mFAST-SeqS Sensitivity of NGS: 8/30 (26.7%) Sensitivity of mFAST-SeqS: 112/121 (92.6%) Aneuploidy was found in 24 patients using mFAST-SeqS only and was correlated with worse prognosis
Ballester et al., 2018 [100]	7	Melanoma	Thirty percent of patients with a negative CSF cytology showed a CSF-ctDNA positivity Droplet digital PCR

NSCLC: non-small-cell lung cancer; CSF: cerebrospinal fluid; PCR: polymerase chain reaction; ctDNA: circulating tumor DNA; BM: brain metastases; LM: leptomeningeal metastases; EGFR: epidermal growth factor receptor; PIK3CA: phosphatidylinositol-4,5-bisphosphate 3-kinase catalytic subunit alpha; HER2: human epidermal growth factor receptor 2; MPL: myeloproliferative leukemia; CDKN2A: cyclin-dependent kinase inhibitor 2A; TKI: tyrosine kinase inhibitor; ALK: anaplastic lymphoma kinase; MET: mesenchymal-epithelial transition factor; SNVs: single nucleotide variants; NGS: next-generation sequencing; iPFS: intracranial PFS; RB: retinoblastoma gene; HR: hormone receptor; ESR1: estrogen receptor type 1;mFAST-SeqS: modified Fast Aneuploidy Screening Test-Sequencing System.

**Table 4:** Studies on LM from NSCLC.

Study	Type of Study	No. of Patients	Treatment	Results
**EGFR TKIs**				
Grommes et al., 2011 [109]	Retrospective	9	Pulsatile high-dose erlotinib (1500 mg weekly)	Radiological response in 6/9 patients (66.7%) Median OS: 12 months
Lee et al., 2013 [110]	Retrospective	25	Arm 1: gefitinib 250 mg/day Arm 2: erlotinib 150 mg/day	Clearance of CSF cytology in 10/25 patients (40%) Erlotinib led to CSF cytology conversion in 64.3% of patients, while only in 9.1% following gefitinib
Yang et al., 2015 [111]	Retrospective	6	Pemetrexed 500 mg/m^2^ day 1; cisplatin 30 mg day 1–2; erlotinib 150 mg day 3–21	Response rate: CR 1/6 (16.6%); PR 2/6 (33.3%); SD 2/6 (33.3%) Median OS: 9 months
Kawamura et al., 2015 [112]	Retrospective	35	Arm 1: high-dose erlotinib (200–600 mg/day every 2–4 days) Arm 2: standard dose erlotinib (150 mg/day)	High-dose erlotinib: radiological response in 3/10 patients (30%), neurological improvement in 6/12 patients (50%) Median OS: high-dose group: 6.2 months Standard dose group: 5.9 months
Jackman et al., 2015 [113]	Phase I	7	2 weeks of high-dose of gefitinib (750–1000 mg/day) and 2 weeks of 500 mg/day	Median OS: 3.5 months Median PFS: 2.3 months CSF cytology clearance in 1/7 patients (14.3%) Neurological improvement in 4/7 patients (57.1%)
Liao et al., 2015 [129]	Retrospective	75	Arm A: Gefitinib + CT Arm B: Erlotinib + CT Arm C: Afatinib + CT Regimen details not available	The association of TKI plus chemotherapy is correlated with prolonged survival in both univariate and multivariate analysis
Tamiya et al., 2017 [114]	Prospective	11	Afatinib 40 mg/m^2^ daily	Median CSF penetration: 2.45% Median CSF concentration: 1.4 ng/mL (2.9 nM) Radiological response: 27.3% Median PFS: 2 months Median OS: 3.8 months
Yang et al., 2017 [115]	Phase I	32	Osimertinib 160 mg daily	20/23 patients (86.9%) had neurological improvement 23/32 (72%) had radiological response
Nanjo et al., 2018 [116]	Prospective	13 (3 definitive LM and 8 possible LM)	Osimertinib 80 mg daily	CSF penetration: 2.5% Median PFS: 7.2 months
Yang et al., 2020 [117]	Prospective	41	Osimertinib 160 mg daily	ORR 62% Median OS 15.2 months
Saboundji et al., 2018 [118]	Retrospective	20	Osimertinib 80 mg daily	100% of patients experienced neurological improvement Median PFS: 17.2 months Median OS: 18 months
Ahn et al., 2020 [119]	Retrospective	22	Osimertinib 80 mg daily	ORR 55% Median OS 18.8 months
Park et al., 2020 [120]	Phase 2	40	Osimertinib 160 mg daily	ORR 55% Median PFS 7.6 months Median OS 16.9 months
Lee et al., 2020 [121]	Retrospective	351 87 with T790 mutation	Osimertinib	No difference in median OS according to T790M mutational status (10.1 months (95% CI 4.3–15.8) versus 9.0 months (95% CI: 6.8–11.21)) Patients treated with osimertinib had a superior OS of 17.0 months (95% CI 15.1–18.9) compared with that of those not treated with osimertinib who had a median OS of 5.5 months (95% CI 4.3–6.6), regardless of T790M mutational status
Ahn et al., 2016 [130]	Prospective	29 (4 with LM)	AZD3759	3/4 patients (75%) had a significant reduction of EGFR expression 1/4 patients (25%) had a CSF conversion in two consecutive samples
Cho et al., 2017 [131]	Prospective	18	Arm 1: AZD3759 200 mg daily Arm 2: AZD3759 300 mg daily	5/18 patients (27.8%) had a radiological response, while 9/18 patients (50%) had a stable disease
Xu et al., 2020 [132]	Prospective	3	erlotinib (150 mg/day) plus nimotuzumab (200 mg/m^2^) weekly	Rapid clinical response within 6–8 weeks from the start of treatment 2/3 patients reported a radiological response
**ALK inhibitors**				
Costa et al., 2011 [133]	Case report	1	WBRT plus crizotinib 250 mg twice daily	PFS: 9 months
Ahn et al., 2012 [134]	Case series	2	Intrathecal MTX plus crizotinib 250 mg twice daily	PFS 5 and 10 months, respectively
Arrondeau et al., 2014 [135]	Case report	1	Ceritinib 750 mg daily	PFS: 5.5 months
Dudnik et al., 2015 [136]	Case series	3	WBRT plus ceritinib 500 mg/daily	PFS patient 1: 18 months; patient 2 and 3: 7 months
Gainor et al., 2015 [137]	Case series	4	Alectinib 600 mg twice daily	Radiological and neurological improvement in 4/4 patients (75%)
Ou et al., 2015 [138]	Case report	1	Alectinib 600–750 mg twice daily	Long-lasting complete response (15 months)
Gainor et al., 2016 [139]	Case series	2	Alectinib 900 mg twice daily	Radiological and neurological improvement for 3.5 and 6 months, respectively
Gaye et al., 2019 [140]	Case report	1	Brigatinib 180 mg once daily with a 7 day lead-in period at 90 mg	PFS 14 months
Pellerino et al., 2019 [141]	Case report	1	Lorlatinib 100 mg once daily	PFS 12 months Complete radiological response
Frost et al., 2020 [142]	Prospective	36 BM 9 LM	Lorlatinib 100 mg daily	Median duration of treatment: 10.4 months PFS: 8.0 months Intracranial response rate: 54% Time to treatment failure: 13.0 months Calculated 12-, 18- and 24-month OS were 65, 54 and 47% TP53 mutations were associated with a shorter PFS (3.7 versus 10.8 months), suggesting a role as strong prognostic biomarker

ALK: anaplastic lymphoma kinase; LM: leptomeningeal metastases; EGFR: epidermal growth factor receptor; TKIs: tyrosine kinase inhibitors; PFS: progression-free survival; ORR: objective response rate; CR: complete response; PR: partial response; SD: stable disease; OS: overall survival; TKI: tyrosine kinase inhibitor; CSF: cerebrospinal fluid; BM: brain metastases; LM: leptomeningeal metastases.

**Table 5:** Ongoing clinical trial on LM (from https://clinicaltrials.gov, last update 1 March 2021).

Study	Number of Patients	Primary Solid Tumor	Treatment	Primary Outcome Measure
NCT04356118 Phase 4	30	NSCLC	Recombinant human endostatin + intrathecal MTX + targeted therapy (EGFR TKIs or ALK inhibitors)	OS Neurological PFS Adverse events
NCT04356222 Phase 4	30	NSCLC	Durvalumab + intrathecal MTX	OS Neurological PFS Adverse events
NCT04315246 Phase 1/2	63	Ductal or lobular BC, NSCLC, melanoma	Intracerebroventricular administration of 177Lu-DTPA-omburtamab	Incidence of AEs and SAEs (time frame: 1 year)
NCT03661424 Phase I	16	HER2-positive BC	Anti-CD3 x Anti-HER2/Neu (HER2Bi) Armed Activated T Cells (BATs)	Type, frequency, severity, duration and timing of AEs Number of patients who achieved the 80% of the total administrations of BATs
NCT04192981 Phase 1	36	Primary solid tumors harboring PIK3CA mutations	GDC-0084 with or without WBRT	Primary: MTD
NCT03974204 Phase: NA	74	BC	---	Proteomic profiles from CSF at diagnosis CSF cytology positivity at diagnosis
NCT04729348 Phase 2	19	Any solid tumors	Pembrolizumab plus Lenvatinib	Six-month OS
NCT04425681 Phase 2	20	EGFR-mutated NSCLC	Osimertinib + bevacizumab	ORR PFS
NCT04148898 Phase 2	80	EGFR-mutated NSCLC	Arm 1: Osimertinib alone Arm 2: Osimertinib + bevacizumab	Intracranial PFS ORR
NCT04233021 Phase 2	113	EGFR-mutated NSCLC	Osimertinib alone	ORR
NCT03696030 Phase 1	39	HER2-positive BC	Intraventricular administration of autologous HER2-CAR T Cells	DLT AEs
NCT03719768 Phase 1b	23	Any solid tumors	Avelumab + WBRT	Safety and DLT
NCT04588545 Phase 1/2	39	HER2-positive BC	Focal RT or WBRT + intrathecal trastuzumab/pertuzumab	MTD OS
NCT02422641 Phase 2	16	Any subtypes of BC	Intravenous high-dose MTX	12-week OS
NCT03613181 Phase 3	150	HER2-negative BC	ANG1005 versus Physician’s Best Choice	OS
NCT03501979 Phase 2	30	HER2-positive BC	Tucatinib + Trastuzumab + Capecitabine	OS
NCT04420598 Phase 2	39	Cohort 5: HER2-positive or HER2-low expressing BC with LM	Trastuzumab deruxtecan	OS

NSCLC: non-small-cell lung cancer; MTX: methotrexate; EGFR TKIs: epidermal growth factor receptor tyrosine kinase inhibitors; ALK: anaplastic lymphoma kinase; OS: overall survival; PFS: progression-free survival; ORR: objective response rate; BC: breast cancer; AEs: adverse events; SAEs: severe adverse events; PK: pharmacokinetics; dOR: duration of objective response; HER2: human epidermal growth factor receptor 2; BATS: bispecific activated T cells; NA: not applicable; HR: hormonal receptor; TNBC: triple-negative breast cancer; QoL: quality of life; CAR: chimeric antigen receptor; WBC: white blood cells; CTCs: circulating tumor cells; DLT: dose-limiting toxicity; RT: radiotherapy; WBRT: whole-brain radiotherapy; MTD: maximum tolerated dose.
